# The Modulatory Effects of Curcumin on the Gut Microbiota: A Potential Strategy for Disease Treatment and Health Promotion

**DOI:** 10.3390/microorganisms12040642

**Published:** 2024-03-23

**Authors:** Junwen Zhu, Lan He

**Affiliations:** College of Bioscience and Biotechnology, Hunan Agricultural University, Changsha 410128, China; helan@stu.hunau.edu.cn

**Keywords:** curcumin, gut microbiota, inflammation, oxidative stress, bioavailability, Intestinal barrier, health

## Abstract

Curcumin (CUR) is a lipophilic natural polyphenol that can be isolated from the rhizome of turmeric. Studies have proposed that CUR possesses a variety of biological activities. Due to its anti-inflammatory and antioxidant properties, CUR shows promise in the treatment of inflammatory bowel disease, while its anti-obesity effects make it a potential therapeutic agent in the management of obesity. In addition, curcumin’s ability to prevent atherosclerosis and its cardiovascular benefits further expand its potential application in the treatment of cardiovascular disease. Nevertheless, owing to the limited bioavailability of CUR, it is difficult to validate its specific mechanism of action in the treatment of diseases. However, the restricted bioavailability of CUR makes it challenging to confirm its precise mode of action in disease treatment. Recent research indicates that the oral intake of curcumin may lead to elevated levels of residual curcumin in the gastrointestinal system, hinting at curcumin’s potential to directly influence gut microbiota. Furthermore, the ecological dysregulation of the gut microbiota has been shown to be critical in the pathogenesis of human diseases. This review summarizes the impact of gut dysbiosis on host health and the various ways in which curcumin modulates dysbiosis and ameliorates various diseases caused by it through the administration of curcumin.

## 1. Introduction

**Figure 1:**
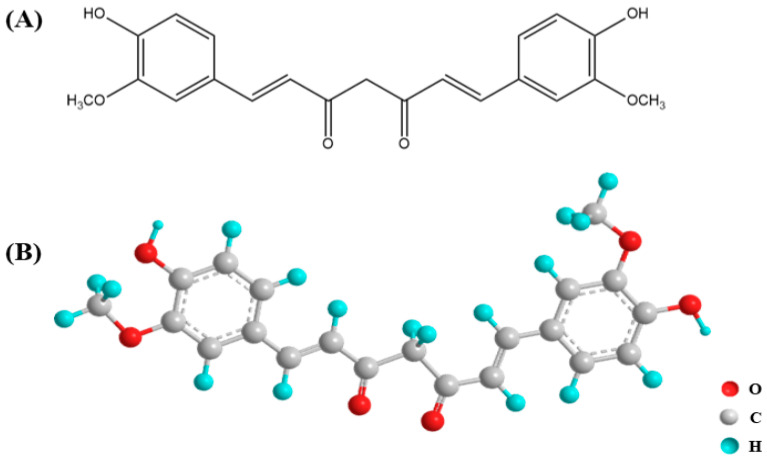
The structure of curcumin. (**A**) The chemical structure formula of CUR and (**B**) the ball-and-stick model of CUR.

Curcumin, 1,7-bis(4-hydroxy-3-methoxyphenol)-1,6-heptadiene-3,5-dione (Figure 1), is a naturally occurring polyphenol extracted from turmeric, a perennial herb that has been widely used for centuries as a dietary spice and also used as a traditional natural medicinal remedy in China and India [1]. Because of the constitutional double bond in its chemical structure, curcumin behaves as an efficacious electron donor, thereby mitigating the generation of redox reactions of reactive oxygen species (ROS) [2], which cause oxidative stress and cellular damage. This property makes curcumin a potent antioxidant widely used in the food supplements industry, among other applications [3]. In addition to its antioxidant properties, curcumin exhibits various other biological activities, such as anti-inflammatory effects, the modulation of lipid metabolism, and the modulation of the immune system [4]. These activities contribute to its potential as an anticancer, antitumor, and antithrombotic agent. Furthermore, curcumin is known to interact with various cellular and molecular targets, including growth factors, chemokines, transcription factors, and cell adhesion molecules, further enhancing its therapeutic potential. However, the content of curcumin in turmeric is very low, and its bioavailability is relatively low as well [5]. Bioavailability refers to the proportion of a drug or compound that enters the bloodstream and reaches the target tissues, thereby influencing its therapeutic efficacy. The limited bioavailability of curcumin poses a challenge to achieving its ultimate therapeutic effect. The contradiction with the low bioavailability of curcumin and its diverse pharmacological activities could be resolved by considering the interactions of curcumin with the gut microbiota. Since this discovery, a large number of clinical trials started to explore the therapeutic potential of CUR for a variety of human diseases, namely cancer, cardiovascular diseases, neurodegenerative diseases, and inflammation (Table 1) [6]. These trials are designed to provide scientific evidence for the efficiency and security of CUR therapies.

The human gut microbiota is considered one of the densest and most active ecosystems of microorganisms and has a crucial function in maintaining human health. It consists of over one billion microorganisms, including bacteria, fungi, viruses, and protozoa [14]. The healthy microbiota comprises four main groups of bacteria, which include Actinobacteria, Firmicutes, Bacteroidetes, and Proteobacteria [15]. These bacteria interact with host receptors, regulate the balance of intestinal flora, and produce metabolites essential for maintaining the function of the intestinal epithelial barrier [16]. This barrier regulates immune function, promotes the metabolism of indigestible carbohydrates, and prevents the invasion of foreign pathogens. Moreover, the intestinal microbiota has a fundamental function in all aspects of the host’s health [17], including nutrient metabolism, protection against pathogens, and the modulation of the gut–brain axis. It directly interacts with the intestinal mucosa and enteric nervous system, contributing to the overall well-being of the host [18]. When the equilibrium of the intestinal flora is disrupted, such as through intestinal infections or dietary changes, it can lead to an increase in intestinal permeability [19]. This disruption promotes the translocation of endotoxins, microbial elements, and microbial metabolites to the circulation, triggering intestinal inflammation and potentially contributing to the progression of various diseases, such as obesity, AS [20], and type II diabetes [21]. The latest research indicates elevated levels of CUR in the gastrointestinal system after the oral intake of curcumin [22]. It has been proposed that this polyphenol interacts dynamically with the intestinal microbiota, modulating the composition and activity and exerting a potential therapeutic effect on diseases caused by the dysbiosis of the intestinal flora [23]. This review aims to thoroughly examine how curcumin modulates the gut microbiota, investigating its viability as a treatment for diseases linked to gut flora imbalance and the bioactivity of curcumin and how it is characterized.

## 2. Curcumin Bioactivity and Characterization

### 2.1. Antioxidation

Oxidative stress (OS) manifests as a disproportion between the generation of reactive oxygen species and the body’s antioxidative protective systems [24]. This disequilibrium might result in cell malfunction and harm, potentially leading to diverse illnesses, such as heart disease, cancer, or diabetes [25]. Studies have suggested that curcumin can act as a bifunctional antioxidant. Firstly, it reacts directly with the active substances, neutralizing them and preventing further damage [26]. Secondly, curcumin induces the upregulation of various cytoprotective and antioxidant proteins [27], enhancing the body’s defense against oxidative stress. Meanwhile, CUR regulates the expression of antioxidant enzymes and thus stabilizes ROS levels mainly through the activation of the cytoprotective protein receptor nuclear factor red lineage derivative 2 (Nrf2) signaling pathway [28]. This transcription factor has a crucial function in cell reactions to oxidative stress by controlling the gene expression for antioxidant enzymes and detoxification proteins, thereby shielding cells from oxidative harm [29]. Under oxidative stress conditions, the oxidation of specific cysteine residues Cys-151, Cys-273, Cys-288, Cys-297, and Cys-257 in Kelch-like ECH-associated protein 1 (Keap1) [30], resulting in the degradation of the Nrf2-Keap1 complex [31], which, in turn, indirectly induces the production of superoxide dismutase (SOD), catalase (CAT), glutathione-S-transferase (GST), glutathione reductase (GR), glutathione peroxidase (GPx), and heme oxygenase 1 (HO-1) [32]. These cytoprotective proteins exert antioxidant activities, protecting cells from oxidative damage.

In addition, curcumin is capable of activating AMP-activated protein kinase (AMPK), a prominent modulator of cellular energy homeostasis [33]. This activation by curcumin helps alleviate oxidative stress-induced damage to the intestinal barrier and mitochondria [34]. The activation of AMPK has also been shown to enhance the transcriptional activity of Nrf2 by phosphorylating Nrf2, thus promoting the expression of Nrf2 target genes [35]. This molecular mechanism helps maintain redox homeostasis and enhance antioxidant capacity.

### 2.2. Anti-Inflammatory

Inflammatory processes are closely related to oxidative stress [36] due to the disproportion between ROS generation and the body’s antioxidant defense, leading to cellular malfunction and an inflammatory reaction [37]. During the progression of cardiovascular disease, the inflammatory reaction manifests as a significant pathological alteration, marked by increased levels of inflammatory indicators like tumor necrosis factor-α (TNF-α), interleukin-6 (IL-6), interleukin-10 (IL-10), C-reactive protein (CRP), monocyte chemotactic protein-1 (MCP-1), or vascular cell adhesion molecule-1 (VCAM-1) [6]. Researchers have found that curcumin is able to attenuate the inflammatory reaction by reducing the levels of pro-inflammatory mediators [38]. This phenomenon could be due to CUR attaching to toll-like receptors (TLRs) and controlling subsequent signaling routes [39], including nuclear factor κ-B (NF-κB), mitogen-activated protein kinase (MAPK), and activator protein 1 (AP-1) [40]. Among them, NF-κB is a main transcription factor that is essential to induce the onset of inflammatory reactions. Curcumin suppresses NF-κB, consequently diminishing the emission of inflammatory agents such as interleukin IL-1β and IL-6 [41]. NF-κB triggers inflammatory diseases primarily through promoting the mobilization and regulation of distinct inflammasomes, which are components of the innate immune system that also regulate the gut microbial composition [42]. The administration of moderate amounts of curcumin effectively inhibited the phosphorylation of NF-κB inhibitory protein (IκB) [43] in a mice model of dextrose sodium sulfate (DSS)-induced colitis, thereby suppressing NF-κB in the intestinal tract [44]. This ultimately attenuated the inflammatory response.

### 2.3. Low Bioavailability

However, curcumin, like most polyphenols, has a relatively low intestinal absorption of CUR after oral administration [45] and is rapidly metabolized in the liver and excreted through the gallbladder, resulting in a very low bioavailability and significant limitations in pathological applications due to its low aqueous solubility and chemically unstable nature [22]. Studies have shown that the highest value of free CUR in the plasma of mice after the oral administration of 0.1 g/kg of curcumin was only 2.25 μg/mL [46]. Nevertheless, if a high dose of curcumin is ingested, its level in plasma is negligible. Some studies have found that the administration of 90–2000 mg/d of curcumin has a more significant effect on ameliorating oxidative stress and inflammation [47,48], whereas in neurodegenerative disorders, 500–2000 mg/d is required [49]. In recent years, in order to improve the bioavailability of CUR, different CUR formulations using a variety of nanocarriers or co-administered with other molecules have been tested to improve their efficacy [45]. But, it has been suggested that the prolonged administration of curcumin can lead to the development of hepatotoxicity, which may be accompanied by the development of gastrointestinal discomfort, skin inflammation, and chest tightness [50]. Therefore, further research is needed to improve the bioavailability of curcumin and to control different dosages for different diseases [24]. It is noteworthy that curcumin mainly acts in the intestine and has a high concentration after oral administration, and we can explore the interaction between curcumin and gut microbiota to improve the bioavailability process of curcumin [51].

## 3. Curcumin Modulates the Gut Microbiota

Studies have shown a preferential accumulation of curcumin within the gastrointestinal tract following either oral or intraperitoneal administration [23]. This accumulation suggests a potential regulatory influence of curcumin on intestinal flora, comprising the abundance, diversity, and constitution of microorganisms [52]. The intestinal flora is commonly accepted to have a role in determining the pharmacological activity of curcumin. Consequently, it is theorized that curcumin directly influences the intestinal flora [22]. This possibly supports the explanation behind the contradiction of the low bioavailability of curcumin compared to the widely described pharmacological benefits of it [53]. The intestinal flora affected by curcumin can influence the absorption, metabolism, and overall therapeutic potential of curcumin.

### 3.1. Curcumin Affects the Abundance of Beneficial Bacteria

More and more studies have proven a close relationship between intestinal dysbiosis and the occurrence of various diseases [54]. Curcumin, known for its modulating effects on bacterial homeostasis [55], has been shown to alter the ratio of beneficial bacteria in the imbalanced gut microbial community, favoring the growth of beneficial bacterial strains [56]. It has been found that the oral administration of curcumin can change the ratio between beneficial and harmful bacteria in the intestinal microbial community [56]. Shen and others found the modulating effect of curcumin on the intestinal microbiota by administering 100 mg/kg to C57BL/6 mice after fifteen consecutive days [53]. The curcumin group showed a significant decrease in the abundance of *Prevotella* and a significant increase in the abundance of *Bacteroidaceae* and *Rikenellaceae* [53]. Various animal model studies have demonstrated that oral curcumin administration increases the abundance of beneficial bacteria like *Bifidobacterium*, *Lactobacillus*, and butyrate-producing bacteria while decreasing the number of bacteria like *Prevotella*, *Bacteroidaceae*, as well as *Rikenellaceae*, which are commonly associated with systemic disease (Table 2) [57]. Furthermore, in a rat model of non-alcoholic fatty liver disease (NAFLD) induced by a high-fat diet (HFD), the addition of curcumin significantly altered the constitution of the gut microbiota compared to other feeding conditions [1]. Additionally, supplementing with curcumin resulted in enhanced liver metabolism, a rise in advantageous bacterial presence, and a decrease in detrimental bacterial strains, linked to dysbiosis caused by a high-fat diet [58,59]. Meanwhile, studies by Zhai and others suggested that curcumin could counteract ochratoxin-induced oxidative damage and lipid metabolism disorders and increase the diversity and abundance of the intestinal flora composition of animal models of liver disease [24], thereby slowing down liver injury. Research indicates that curcumin enhances the presence of advantageous bacterial varieties, and targeting its impact on gut microbiota composition could be beneficial for treating various diseases [60].

### 3.2. Curcumin Affects the Intestinal Barrier

**Figure 2:**
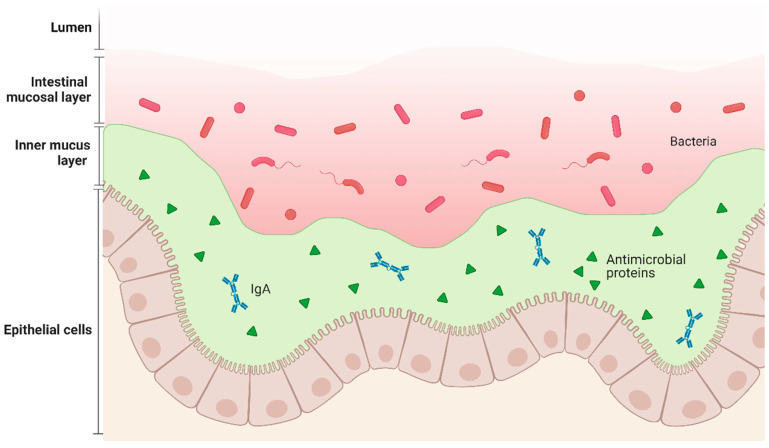
Structure of the intestinal barrier. The first layer is lumen. The second layer is the intestinal mucus layer that contains bacteria and serves as a defense barrier against harmful bacteria. On the third layer is the inner mucus layer which is tightly bound to the lower epithelial cells and has antimicrobial peptides to maintain the intestinal barrier function.

CUR not only affects the component of the intestinal microbiota but also strengthens the intestinal barrier. The intestinal barrier comprises four distinct types of lamins (Figure 2) [69]. Initially, the primary layer contains the enzyme alkaline phosphatase (IAP). IAP possesses the capability to neutralize the bacterial endotoxin lipopolysaccharide [70]. Research indicates that administering curcumin orally can increase IAP activity threefold and reduce the levels of circulating endotoxin lipopolysaccharide (LPS) [71], thereby directly demonstrating curcumin’s regulatory impact on the intestinal barrier’s initial layer [72]. The intestinal mucosal layer, constituting the second layer, is crucial in separating luminal contents from epithelial cells and preventing pathogenic bacteria from entering. With the disappearance of the second layer [73], intestinal epithelial cells would directly interact with luminal bacteria, resulting in intensified intestinal inflammation. The rise in intestinal acidic mucins [74], driven by curcumin, enhances synthesis and minimizes the breakdown of the intestinal mucosal layer, preserving its structure [75]. The third stratum is made up of close connections among intestinal epithelial cells, which block the transfer of detrimental substances like foreign antigens, microbes, and toxins from the intestinal cavity, simultaneously allowing vital nutrients, electrolytes, and water to flow from the intestinal cavity into the bloodstream [69]. Through trans-epithelial and both trans- and paracellular transportation, a defense mechanism against bacterial endotoxins is established, aiding in preserving the intestinal barrier’s integrity [76]. Antimicrobial peptides, found in the last layer, prevent bacteria from breaching the intestinal barrier [77]. α-defensins and β-defensins possess bactericidal properties, with α-defensins being significantly influential within the body. This factor controls the makeup of the intestinal microbiota [69]. Research indicates that curcumin enhances the production of antimicrobial peptides [78]. The quartet of layers collaboratively functions to preserve the operational steadiness of the intestinal barrier. Nonetheless, studies have discovered that disrupted intestinal morphology results in intestinal oxidative stress damage and ROS accumulation and induces apoptosis in intestinal epithelial cells [79]. Also, if the intestinal barrier integrity is compromised, it causes intestinal epithelial cells and localized chronic inflammation [80]. Persistent inflammation is acknowledged as a possible factor in the emergence of conditions like atherosclerosis and diabetes. Research indicated that curcumin markedly slowed down atherosclerosis development and glucose intolerance [37] by lowering the levels of endotoxic lipopolysaccharides in the bloodstream, which are triggered by a Western diet. Curcumin’s role in safeguarding the functionality of the intestinal barrier is noteworthy [80].

## 4. Dysregulation of Gut Microbial Ecology and Related Diseases

### 4.1. Disorders of Lipid Metabolism

The gut microbiota plays a key role in controlling various metabolic activities, including the balance of host energy, the processing of glucose, and the metabolism of lipids [19]. Numerous research findings indicate that metabolic disorders frequently accompany ecological imbalances in gut flora, hinting at a tight link between gut microbial activity and prior metabolic stabilization [81]. The hypothesis suggests a connection between dysbiosis in intestinal flora and lipid metabolism disorders, entailing the creation and breakdown of lipids into fatty acids, triglycerides, and cholesterol [82]. Nutritional habits, significantly impacting gut microflora variety, can modify gut microbiota composition via diets rich in sugar and fat, directly contributing to lipid metabolism issues [83]. Furthermore, alterations in intestinal flora composition exert an influential factor in the maintenance of intestinal epithelial cells and intestinal health, influencing liver lipid metabolism to enhance lipid oxidation, controlling lipid accumulation in fat tissue, and mitigating metabolic conditions associated with obesity and NAFLD [84]. Significantly, research indicates that various fat sources impact the gut microbiota’s composition in distinct ways. A comparison was made between mice on a high-fat diet abundant in saturated fat lard and those on a calorie-dense, HFD-like n-3 PUFA-rich fish oil [85], revealing that the phylogenetic variety in lard-diet mice matched that of the advantageous bacteria *Akkermansia muciniphila*, *Lactobacillus*, and *Bifidobacterium* [86]. Presently, an increasing number of research works have shown that focusing on the gut microbiota enhances human metabolic processes [87], necessitating deeper exploration into how lipids metabolize and microbes function beforehand.

#### Obesity

The development of obesity is closely related to the alteration in the constitution of and reduction in the diversity of the gut microbiota [88]. It has been proposed that the composition of the intestinal microbiota varies with body mass index (BMI), and at lower BMI, the content of thick-walled phyla increases in the host body [89], especially the number of Actinobacteria phyla, while the level of Bacteroidetes phyla is significantly reduced [90], which leads to a decrease in the Bacteroidetes phyla/thick-walled phyla ratio (B/F) in the obese population [91]. In addition, it has been suggested that gut microbes can regulate the absorption of simple sugars in the intestinal lumen and modulate hepatic lipogenesis in order to modulate obesity [91]. One study also found that lipopolysaccharides make a crucial difference in the pathogenesis of obesity. In an obese mouse model, circulating LPS concentrations were found to be 2–3 times higher than in normal healthy mice [92], thus suggesting that the gut microbiota may be a central factor in the stabilization of obesity. Moreover, the gut microbiota regulates microglia, a type of immune cell located in the brain, by activating microglia and neuroinflammation and by acting on hypothalamic neurons, thereby reducing food intake while increasing energy expenditure and ameliorating obesity [93]. More interestingly, gut flora metabolites activate enteroendocrine cells to release advocacy hormones that interact directly with the enteric nervous system and its innervating vagus nerve [94], which produces localized signals that can influence appetite and satiety and reduce body weight.

### 4.2. Immune System

Maintaining the gut microbiota’s ecological balance is vital for regulating immune reactions [19]. It has been shown that the dysregulation of the intestinal microbiota and its associated metabolites has a major effect in rupturing intestinal integrity [16], which affects immune homeostasis and thus the function of peripheral tissues. LPS is present in the outer membrane of Gram-negative bacteria, which is the most abundant bacterium in the intestinal flora, and the lipid A component of LPS is mainly bound to the toll-like receptor TLR4 [95], which is expressed in macrophages, enterocytes, and endothelial cells (ECs), among others, triggers various signaling routes, and encourages the secretion of pro-inflammatory agents [96] and the expression of chemokines and leukocyte adhesion molecules, leading to the development of chronic inflammation [97]. In a study that identified compositional differences in the intestinal microbiota in a mice model of inflammatory bowel disease (IBD), there was a decrease in the overall diversity of the gut flora, a decrease in the number of thick-walled phyla, with an increased proportion of actinomycetes and ascomycetes [98]. In particular, there was a rise in pro-inflammatory species of Escherichia and Fusobacterium, alongside a reduction in anti-inflammatory species of Roseburia and Faecalibacterium [98]. In addition, this study proposes that the functional immune barrier of the intestine is mainly located below the physical barrier of intestinal epithelial cells. Intestinal epithelial cells detect bacteria and other microorganisms through TLR4 and other pattern recognition receptors (PRR) expressed on immune cells, such as macrophages and DC cells [99]. These receptors identify pathogen-associated molecular patterns (PAMPs) on microorganisms, resulting in a normalized immune response [19].

#### 4.2.1. Atherosclerosis

Several research studies have indicated that the dysbiosis of intestinal flora could lead to the development and evolution of atherosclerosis [100]. The birdshot sequencing of the intestinal macrogenome has shown that the intestinal microbial community is different in individuals with atherosclerosis than in healthy controls [101]. Furthermore, it has been shown that healthy intestines and intestinal segments with atherosclerotic lesions contain DNA from different bacterial species in the same individual, with higher abundances of the Enterobacteriaceae and Streptococcus species in the diseased segments [102], which suggests that alterations in the constituent of the intestinal microbiota are a potential pathogenetic mechanism for AS. The ecological dysregulation of the gut microbiota increases intestinal permeability and promotes the LPS/TLR4-mediated release of inflammatory factors [40], which leads to the adhesion of monocytes to the endothelial layer to form foam cells, namely macrophages, and ultimately contributes to the formation of atherosclerotic plaques. In addition, ecological dysregulation leads to alterations in various metabolic pathways Trimethylamine N-Oxide (TMAO) is an intestinal-derived colony-associated metabolite [103], and it has been demonstrated that the production of TMAO is mainly derived from Trimethylamine (TMA) produced by substances such as l-carnitine and dietary phosphatidylcholine [104], which is transported through the portal vein into the liver where it can be absorbed into the bloodstream by the Flavin TMA is further oxidized to TMAO by Flavin containing monooxygenase 3 (FMO3), which has been shown to be an important risk factor for the development of atherosclerosis [104]. Disturbed intestinal flora contributes to TMAO production and increases foam cell formation, and TMAO promotes the progression of AS by inhibiting reverse cholesterol transport (RCT) [105].

#### 4.2.2. Inflammatory Bowel Disease

**Figure 3:**
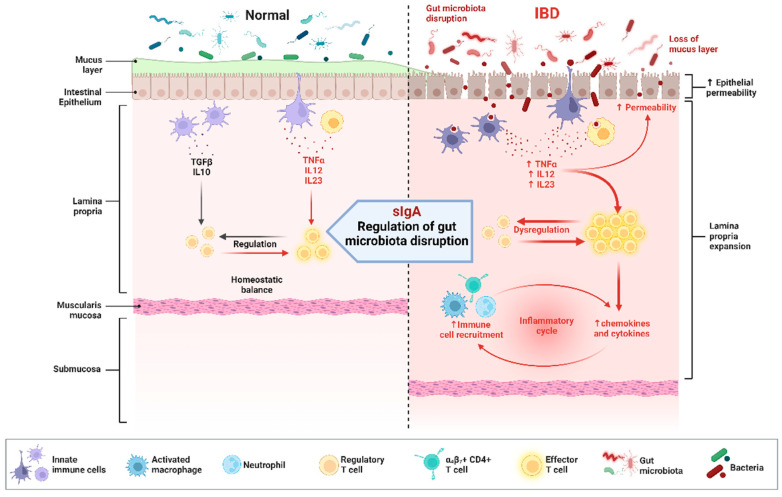
Comparison of IBD with healthy gut and immune response to IBD. The disturbance of the gut microbiota and the damage of the intestinal barrier result in an increase in the permeability of the intestinal epithelial cells, which allows the entry of foreign harmful bacteria into the intestinal lumen, causing a massive release of pro-inflammatory factors and disrupting the immune system, and inducing the development of IBD. Nevertheless, the imbalance of intestinal flora can be regulated through the secretion of sIgA in the intestinal lumen, improving the outcome of IBD.

The main manifestations of inflammatory bowel disease are Crohn’s disease (CD) and ulcerative colitis (UC) [106]. Several studies already demonstrated that the occurrence and development of IBD are impressed by various factors such as the immune system and gut microbiota and that the composition of the gut flora in IBD patients is significantly different from that in healthy humans [107], suggesting that the constituent and ecological stability of the intestinal microbiota is a key factor in inducing the pathogenesis of IBD [108]. It has been proposed that the abundance of beneficial bacteria like *Bifidobacterium bifidum*, *Fusobacterium rectum*, and *E. pumilus* was significantly reduced in the gut flora of patients with UC, whereas the level of *Mimicobacterium* fragilis was elevated [98]. It has also been proposed that other harmful bacteria were growing rapidly in UC. Meanwhile, some studies suggested that the potential pathogenesis of intestinal microbiota-induced IBD may be linked to the functional impairment of the gut barrier by immune cells [109], which is impaired, leading to increased exposure to luminal microorganisms and promoting inflammatory responses [110]. Immunoglobulin A (IgA) is the most common antibody subtype in the gut that binds to intestinal microbes, and since IgA is a specific component of breast milk [111], another study thus proposed that feeding through breast milk protects neonates from colitis. Secondly, IgA is transported on host epithelial cells via the polyclonal immunoglobulin receptor (pIgR) and released into the intestinal lumen as secretory IgA (sIgA) [112]. It has been demonstrated that sIgA as a crucial effect in the ecological stability of the intestinal microbiota. Also, when the secretion of sIgA is inhibited, it can lead to the disruption of the bacterial flora, which can further result in damage to the intestinal barrier (Figure 3) [113]. Certain harmful bacteria, such as Klebsiella pneumoniae, are able to interact with macrophages, leading to the release of pro-inflammatory cytokines, like IL-1β and TNF-α, and the activation of inflammatory signaling pathways [114], which promotes the onset and development of intestinal inflammation. Therefore, maintaining the stability of intestinal flora and improving the intestinal barrier function may be an important method to treat IBD in the future.

## 5. Curcumin in the Treatment of Related Diseases

### 5.1. Curcumin Improves Obesity

More and more evidence suggested that the composition of the gut microbiota is closely linked to the pathogenesis of obesity [115]. Under the conditions of obesity, the ratio of Firmicutes to Bacteroidetes in the gut is elevated. However, after the administration of an effective dose of CUR, a significant reduction in the F/B ratio was observed. This reduction was accompanied by a decrease in the number of *Lachnospiraceae* and *Ruminococcaceae*, as well as an increase in the abundance of *Bacteroides*, *Riskenellaceae*, and *Prevotellaceae* within the Bacteroidetes. These changes resulted in a significant reduction in the F/B ratio and alleviation of excessive accumulations of short-chain fatty acids (SCFAs) [116]. Bacteria such as *Bifidobacterium*, *Lactobacillus* and *Akkermansia muciniphila* have been reported to potentially play a key role in anti-obesity in animal models and humans [117]. This was also confirmed by the finding of significantly increased abundance of *Bifidobacterium* and *Akkermansia* spp. in a targeting study of beneficial intestinal bacteria in HFD-induced obese mice [118].

Furthermore, some studies have confirmed that curcumin performs several biological functions in different organs, including adipose tissue [119]. First, curcumin may have an effect on adipose tissue production [120]. Many researchers suggested that curcumin inhibits mitogen-activated protein kinase activities such as ERK, JNK, and p38, thereby inhibiting 3T3-L1 adipocyte differentiation [121]. CUR may also inhibit adipogenic genes by suppressing the expression of the lipogenic genes peroxisome proliferator-activated receptor gamma (PPARγ) and C/EBP alpha [122]. The administration of an effective dose of curcumin reduces obesity by decreasing ependymal adipose tissue [123], increasing energy expenditure, and decreasing lipid accumulation in the body, as well as decreasing adipose tissue inflammation by avoiding phagocytic infiltration into the adipose tissue and increasing lipocalin production [124], as demonstrated in a mouse obesity model triggered by a diet rich in fat and sugar. A research study found that administering 0.2 g/d of CUR to obese mice notably decreased white fat in the group given curcumin unlike the high-fat group [125]. Secondly, in a clinical trial of curcumin for the treatment of obesity, it was found that after the administration of curcumin, BMI was normalized in overweight people, while the serum triglyceride level was significantly reduced, and in the liver [126]. CUR enhanced the HFD-induced insulin sensitivity, blocked lipogenesis, and achieved the effect of the action of the treatment of obesity [9]. In addition, CUR may indirectly maintain cellular homeostasis by regulating the expression and activity of lipid transporter proteins, which are responsible for cholesterol uptake and efflux [127].

### 5.2. Curcumin Cures Atherosclerosis

Atherosclerosis significantly contributes to the development of conditions like coronary heart disease, cerebral infarction, and peripheral vascular disease. In the intestines of AS patients, an increased abundance of Firmicutes with Aspergillus phylum at the phylum level was found to be associated with plaque formation and instability [101,128]. At the genus level, the number of *Bifidobacterium*, which exerts a beneficial bacterial role, decreased, whereas the relative abundance of harmful bacteria, such as *Klebsiella* and *Escherichia*, increased [129]. At the same time, it was proposed that CUR was able to alter the F/B ratio in the gut, increasing Bacteroidetes as well as probiotics such as *Bifidobacterium* and *Lactobacillus*. As the relative abundance of *Bifidobacterium* increased, it was able to break down indigestible dietary fiber in the gastrointestinal tract into SCFAs and enhance the intestinal barrier through AMPK [130].

**Figure 4:**
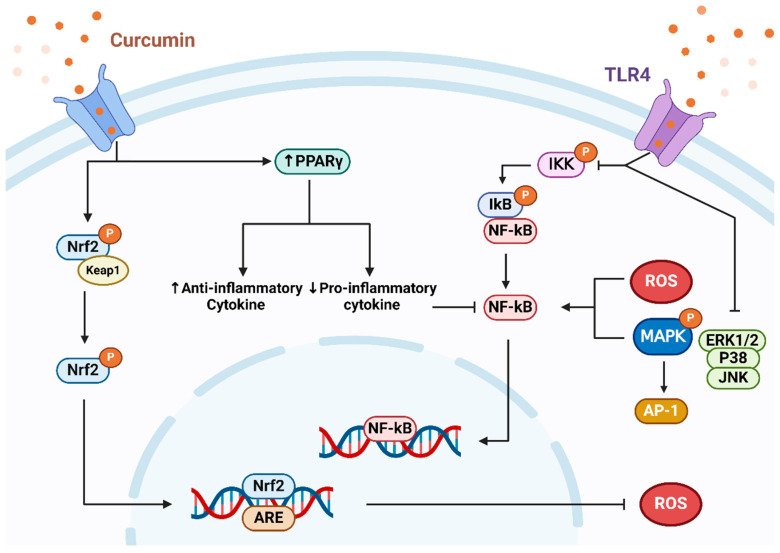
Curcumin inhibits oxidative stress and ameliorates inflammatory pathways. CUR can promote the disassembly of the Nrf2-Keap1 complex and activate the expression of Nrf2/ARE transcripts to exert its antioxidant effect. CUR also regulates inflammatory factors through the upregulation of PPARγ, which indirectly inhibits NF-κB expression. However, CUR was able to inhibit NF-κB transcription directly with TLR4 receptor mediated inhibition, and TLR4 directly regulated the MAPK signaling pathway to inhibit the release of pro-inflammatory factors, which ultimately achieved the purpose of alleviating inflammation.

In addition, it is closely related to oxidative stress and local inflammatory response [101]. Curcumin was able to increase the relative abundance of *Bifidobacterium genera*, and thus *Bacteroides vulgatus* and *Bacteroides dorei*, by decreasing LPS production, improving intestinal barrier permeability, inhibiting pro-inflammatory immune responses, and delaying AS formation [131]. Numerous research findings indicate that curcumin contributes to decelerating atherosclerosis progression by obstructing the toll-like receptor (TLR4)-mediated signaling route [132]. TLR4, serving as a signaling receptor, is crucial in plaque development, a principal factor in the progression of atherosclerosis. Inhibiting the TLR4 signaling pathway not only hinders the activation of NF-κB and MAPK [133] but also diminishes the emission of inflammatory cytokines and reactive oxygen species, achieving anti-inflammatory and antioxidant outcomes. In addition, the inhibition of TLR4 was able to modulate macrophage depolarization, regulate the TLR4/MAPK/NF-κB pathway in macrophages (Figure 4) [1], and reduce the secreting of interleukin IL-4 or IL-13. CUR possesses the ability to counteract inflammation and attenuate the development of atherosclerosis by inhibiting TLR4 expression, as demonstrated in animal model studies [134]. Furthermore, scientists suggest that curcumin’s ability to suppress TLR4 expression might be linked to its role in hindering the NADPH-driven production of reactive oxygen species inside cells [135]. Interestingly, several research studies have pointed out that the therapeutic effect of curcumin on atherosclerosis may depend on different curcumin doses. A meta-analysis revealed that curcumin at a dose of 100–200 mg/kg per day had the best therapeutic effect on atherosclerosis, but doses exceeding 200 mg/kg reduced the positive effects of curcumin [136], which may be linked to the lower bioavailability of curcumin. However, the optimal dose of curcumin to be administered still needs to be confirmed by further studies.

### 5.3. Curcumin Relieves IBD

Inflammatory bowel disease (IBD) occurrence is closely related to the stability of intestinal flora. In an animal model of IBD, supplementation with appropriate amounts of curcumin was found to increase the relative abundance of *Lactobacillus* [137], which were able to enhance mucosal immunity and improve the intestinal barrier function in mice by increasing sIgA, an immunoglobulin that plays an important role in improving intestinal microbial disorders. IBD is also a result of the upregulation of TLR4/NF-κB/AP-1 signalling driven by IBD [99]. In a rodent model of trinitrobenzene sulfonate (TNBS)-induced colitis, curcumin has been suggested to ameliorate inflammation by reducing TLR4 signaling [138]. CUR inhibits LPS immunity by binding to the extracellular TLR4 structural domain-bound protein, myeloid differentiation protein 2 (MD-2), which inhibits the LPS immune response and reduces the release of inflammatory factors [36]. More importantly, the NF-κB is a major contributor to the pathogenesis of IBD [139], and it has been demonstrated that curcumin could block NF-κB expression by regulating the NF-κB/IκB pathway [140]. CUR interferes with the upstream signaling of the IκB kinase, prevents IκB degradation, and inhibits the activation of NF-κB by reducing the expression of TNF-α, IL-1, IL-6, and other cytokines release [141], thereby reducing inflammatory response. Meanwhile, researchers have researched into and understood the severity of intestinal inflammation and the amount of NF-κB p65 [142], which was found to contain higher amounts in the intestines of patients with enterocolitis. Curcumin was able to lower the level of TNF-α expression [143] while significantly reducing nitric oxide (NO) production, thereby inhibiting oxidative stress and exerting beneficial effects on IBD. Secondly, it was shown that curcumin was able to inhibit inflammation by selectively blocking the Cyclooxygenase-2 (COX-2) receptor [144]. In a rat model induced by TNBS for a sustained period of two weeks, the administration of an effective dose of curcumin could inhibit iNOS/COX-2 expression and attenuate the activation of p38 MAPK [106], which has an important function in the regulation of the transcription and release of inflammatory factors.

## 6. Conclusions and Prospects

Currently, the composition, diversity, and ecological stability of the gut microbiota are of crucial importance in delaying and ameliorating the occurrence and progressive development of many diseases [145]. The dysbiosis of the intestinal flora can cause lipid metabolism disorders, immune system malfunction, and an intact intestinal barrier can prevent the invasion of harmful bacteria from entering the host’s internal body circulation, but the specific mechanisms by which alterations in the microbiota in the body affect the host’s health have not yet been fully determined [146]. However, it has been found that oral curcumin supplementation is able to target improvements in gut barrier function and that higher levels of CUR residues have been found in the gut after oral CUR administration [147], emphasizing the possible positive effects of CUR on the gut microbiota [148]. The important biological activities of curcumin, such as anti-inflammatory, antioxidant, anti-obesity, and anticancer, have long been demonstrated [149], and it is able to fully utilize its pharmacological effects through various molecular targets [150]. The anti-inflammatory effect of CUR is mainly achieved by inhibiting the NF-κB, reducing the release of a variety of pro-inflammatory factors [151], accompanied by the scavenging of free radicals and the downregulation of ROS generation, thereby reducing oxidative stress and exerting its antioxidant effects [152]. Therefore, CUR can improve the occurrence and development of atherosclerosis and inflammatory bowel disease [136]. In addition, CUR maintains intestinal barrier function by regulating multiple signaling pathways to prevent damage from dietary factors or endogenous injury [80]. Secondly, CUR can regulate lipid metabolism and production through MPAK signaling, thus achieving the goals of treatments for obesity [153]. Despite curcumin’s low systemic bioavailability, CUR has received widespread attention for its multiple pharmacological therapeutic uses [22], so curcumin may provide benefits by acting on the gut microbiota, but the specific mechanism of its modulation of the gut microbiota composition to achieve targeted treatments of multiple diseases still requires more research to provide a theoretical basis.

## Figures and Tables

**Table 1:** The biological activity of curcumin plays a therapeutic role in various diseases. MCP-1—monocyte chemoattractant protein-1, IL-1β—interleukin-1β, IL-4—interleukin-4, VGEF—vascular endothelial growth factor, HDL—high-density lipoprotein, BMI—body mass index.

Diseases	Curcumin Dose	Duration	Effects	Reference
Metabolic syndrome	1 g/day	8 weeks	↓TNF-α, IL-6 and MCP-1	[7]
Obesity	1 g/day	4 weeks	↓IL-1β, IL-4, VGEF	[8]
Diabetes	500 mg/day	15–30 days	↓ Oxidation	[9]
Atherosclerosis	0.5 g/day	7 days	↓ Serum lipid peroxides and serum total cholesterol levels ↑ HDL cholesterol	[10]
Colorectal cancer	1.08 g/day	10–30 days	↓ TNF-α ↑ p53 expression and improve BMI	[11]
Rheumatoid arthritis	1.2 g/day	2 weeks	Improvement in joint swelling, morning stiffness	[12]
Fatty liver disease	1 g/day	8 weeks	↓ BMI ↑ Liver function	[13]

**Table 2:** The contributions at the phylum, family, and genus levels.

Levels		Functions	Reference
Phylum	Firmicutes	Produces beneficial SCFAs Maintains balanced gut flora Supports intestinal barrier integrity	[61,62,63,64]
	Bacteroidetes	Secretes antimicrobial substances Balances nutrients in the gut Supports immune system functioning	
	Proteobacteria	Produces beneficial SCFAs	
	Actinobacteria	Promotes bioactive substances Inhibits pathogenic bacteria Produces acetic acid and butyric acid	
Family	*Bacteroidaceae*	Breaks down polysaccharides Aids absorption	[61,62,63,64,65,66]
	*Prevotellaceae*	Produces acetic acid	
	*Bifidobacteriaceae*	Maintains intestinal microbial balance	
	*Lactobacillaceae*	Secretes sIgA produces acids	
	*Enterobacteriaceae*	Produces beneficial SCFAs	
	*Micromonosporaceae*	Supports immune system health	
Genus	*Bacteroides*	Secretes sIgA	[61,62,63,64,65,66,67,68]
	*Prevotella*	Produces beneficial SCFAs	
	*Bifidobacterium*	Maintains flora balance	
	*Escherichia*	Involved in degradation and fermentation of proteins and fibers	
